# Differential apoptosis gene expressions of rhabdomyosarcoma cells in response to enterovirus 71 infection

**DOI:** 10.1186/1471-2334-12-327

**Published:** 2012-11-28

**Authors:** Weifeng Shi, Xiang Li, Xueling Hou, Hongjun Peng, Qingbo Jiang, Mei Shi, Yun Ji, Xiping Liu, Jinbo Liu

**Affiliations:** 1 Department of Clinical Laboratory, The Third Affiliated Hospital of Suzhou University, Changzhou, Jiangsu, 213003, China; 2 Department of Orthopedics, The Third Affiliated Hospital of Suzhou University, Changzhou, Jiangsu, 213003, China

**Keywords:** Enterovirus 71, PCR array, FasL, CD40L, Apoptosis

## Abstract

**Background:**

Enterovirus 71 (EV71) infection can induce the apoptosis of infected cells. The aim of this study is to explore the effect of EV71 infection on apoptosis mechanisms in virus-infected human rhabdomyosarcoma (RD) cells.

**Methods:**

The apoptosis of RD cells was examined using annexin V-FITC/PI by flow cytometry and cytokines were detected by ELISA. Cellular RNA was extracted and transcribed to cDNA. PCR array was employed to analyze the expressions of 84 apoptotic genes from EV71-infected RD cells at 8 and 20 h postinfection, respectively. In addition, the expressions of FasL, caspase, AKT2, JNK1/2, c-Jun and NF-κB proteins were detected by western blotting.

**Results:**

Flow cytometry demonstrated that the apoptosis or death of EV71-infected RD cells was increased by 37.1% with a multiplicity of infection (MOI) of 5 at 20 h postinfection. The production of IL-4, IL-10 and TNF-α was enhanced by the subsequent EV71 infection. PCR array revealed significant changes in the expressions of apoptotic genes. Among 84 genes, 42 genes were down-regulated after EV71 infection at 8 h, whereas 32 genes were up-regulated at 20 h postinfection. Moreover, the ligands of TNF superfamily such as FasL, CD40L and TNF-α were significantly up-regulated and enhanced the expressions of apoptosis-related cysteine peptidases, including caspase-10, -8, -7 and -3. In addition, EV71 infection induces the phosphorylation of AKT2, JNK1/2, c-Jun and NF-κB at 20 h postinfection.

**Conclusion:**

PCR array for the determination of apoptosis gene expressions is an informative assay in elucidating biological pathways. During the early stage of EV71 infection, the apoptotic process of RD cells is significantly delayed. EV71 infection can also induce the expressions of FasL, TNF-α and CD40L, which contribute to the apoptosis of RD cells.

## Background

EV71 is a member of *Picornaviridae* family composed of a large number of small non-enveloped, positive strand RNA viruses with a genome size of approximately 7.4 kb [1,2]. Both EV71 and coxsackievirus A16 (CVA16) belong to the human enterovirus A species, which are major causative agents causing hand, foot, and mouth disease (HFMD) in children [3]. However, patients infected with EV71 are liable to cause aseptic meningitis, encephalomyelitis, pulmonary edema and death [4,5]. EV71 was first identified in 1969 in California when it was isolated from the feces of an infant suffering from encephalitis [6]. Subsequently, EV71 infection is widely popular in many countries and regions, such as Taiwan, Singapore, Malaysia, and Hongkong, as well as mainland China [7-11]. Up to now, the molecular pathogenesis of EV71 infection is still elusive.

Apoptosis is essential for the maintenance of homeostasis in the immune system, which is characterized morphologically by internucleosomal DNA cleavage, chromatin condensation, membrane blebbing, cell shrinkage, apoptotic body formation and cell death. The process may be triggered by interactions of pro-apoptotic stimuli coupled with various factors such as death receptors, mitochondrial apoptotic pathway and endoplasmic reticulum stress [12,13]. In an attempt to prevent viral replication, dissemination or persistent infection of cells, many protective measures are actually involved in the induction of apoptosis that is the host response to curtail the reproductive cycle of the virus through premature lysis [14,15]. In addition, the apoptosis of host cells can facilitate macrophages to phagocytose dead cells for preventing dysregulated inflammatory reactions or initiating specific immune responses in the infected host [16].

In order to elucidate the molecular basis of the host response to viral infection, anti-apoptotic response is essential for identifying the targets to reduce cell or tissue damage resulting from inflammatory responses. As described previously, EV71 can induce the apoptosis of human endothelial cells, T lymphocytes and nerve cells [17-19]. However, little information is known about the mechanisms of RD cell apoptosis by EV71 infection. In this study, PCR array was used to detect 84 genes associated with apoptosis and explore the host response at different time points after EV71-infection in RD cells as well as molecular mechanisms of apoptosis.

## Methods

### Antibodies and chemicals

Dulbecco's modified Eagle's medium (DMEM) and fetal bovine serum (FBS) were purchased from Thermo Scientific HyClone (UT, USA). Anti-caspase-8, -3, AKT2, JNK1/2, c-Jun and NF-κB p65 rabbit polyclonal antibodies were purchased from Signalway Antibody (Pearland, TX, USA). Rabbit polyclonal phospho-specific antibodies, including p-JNK1/2, p-AKT2, c-Jun and p-NF-κB p65 antibodies were also from Signalway Antibody. Anti-caspase-10, -7, and FasL were from Cell Signaling Technology (Beverly, MA, USA). Goat anti-glyceraldehyde-3-phosphate dehydrogenase (GAPDH), β-actin antibodies and IgG secondary horseradish peroxidase (HRP) conjugated were from Signalway Antibody.

### RD cell culture and EV71 infection

RD cells were purchased from CBTCCCAS (Chinese Academy of Sciences Cell Bank of Type Culture Collection) and cultured in high glucose DMEM supplemented with 10% FBS at 37°C in a humidified incubator with 5% CO_2_. When cells reached up to 90% confluence, the medium was removed and the monolayer cells were washed once with PBS. One batch of uninfected RD cells in 25 cm^2^ culture flask were used as the control, while another two batches of RD cells were infected with UV-inactivated EV71 strain CCTCC/GDV083 (ATCC VR-784) (China Center for Type Culture Collection, CCTCC) and alive EV71 strain GDV083 at an MOI of 5 in a 4 mL of virus inoculum diluted with maintenance medium. Approximately 1 × 10^6^ cells were incubated with EV71 at an MOI of 5 or as indicated and allowed to absorb for 2 h at 37°C. Unbounded viruses were removed by washing the cells with medium, and 15 mL of maintenance medium was added. Infected cells and culture supernatants were collected at different time intervals. Virus titers in both supernatant (extracellular) and cell lysate (intracellular) were determined using plaque assays, as described previously, at the designated time points [20].

### Viability of RD cells

The viability of RD cells was assayed using the trypan blue exclusion method. Cells were cultured with EV71 at an MOI of 5. After different incubation periods, cells were re-suspended in trypan blue solution and counted under a light microscope. RD cells without being stained by trypan blue were counted as the viable cells. The viability of RD cells was calculated through the total number of cells dividing by the number of viable cells.

### Evaluation of apoptosis in RD cells by annexin V-FITC/PI binding assay

RD cells were seeded at a density of 1 × 10^5^ cells onto pre-treated coverslips in a 6-well plate. After incubation at 37°C overnight, the cells were inoculated at an MOI of 5 with EV71 strain and UV-inactivated EV71 strain for 2 h. After absorption, the inoculum was removed, and replaced with 3 mL of maintenance medium. The cells were subjected to incubation for another 20 h, and then fixed with 3.7% paraformaldehyde for 30 min at room temperature. The coverslips were stained by annexin V- fluorescein isothiocyanate (FITC) / propidium iodide (PI) (Vector Laboratories, Burlingame, CA, USA) and observed under a fluorescence microscope. Flow cytometry was performed with Vybrant apoptosis assay kit (Molecular Probes, Eugene, OR) according to the manufacturer’s instructions. A total of 2 × 10^4^ RD cells were infected with EV71 and harvested at 8 and 20 h postinfection. Following washes in cold phosphate-buffered saline (PBS) and re-suspension in 195 μL of 1× annexin-binding buffer, 5 μL annexin V- FITC and 10 μL PI were added to the cells and incubated at room temperature for 15 min. Stained cells were then analyzed by flow cytometry using FACS Canto System (BD Biosciences, San Jose, CA). Normal living cells were not stained with annexin V-FITC and PI, while cells stained with annexin V-FITC alone were defined as the early stage of apoptosis and the cells stained with both annexin V-FITC and PI were defined as the late stage of apoptosis or necrosis. Quantitative analysis was conducted by determining the percentage of stained cells among total cells.

### ELISA for cytokines

RD cells were infected with EV71 at an MOI of 5 for 2 h at 37°C. Infected RD cells were washed 2 times and cultured in DMEM medium. Culture supernatants were harvested at 0, 8, 20 and 32 h after EV71 infection. The expression levels of IL-4, IL-10 and TNF-α were measured by ELISA kits (R&D System, USA) according to the manufacturer’s protocols. All experiments were repeated three times.

### Total RNA preparation and PCR array

After incubation at 37°C for 8 and 20 h, both uninfected and infected RD cells were harvested for the extraction of total RNA. Total RNA was extracted by SV total RNA isolation system (Promega, Madison, WI, USA). A total of 1 mg RNA for each sample was used for reverse transcription through RT-PCR Kit (catalog#CTB101; CT biosciences, China) on the ABI 9700 thermocycler (ABI, Foster City, CA). PCR arrays were performed with customized PCR containing pre-dispensed primers (CT biosciences, China) on the LightCycler 480 (Roche Diagnostics, Mannheim, Germany) using SYBR MasterMix ((catalog#CTB101; CT biosciences, China). Each PCR contained 10 ng of synthesized cDNA. The thermocycler parameters were performed with an initial denaturation at 95°C for 5 min followed by 40 cycles of denaturation at 95°C for 15 s, annealing at 60°C for 15 s and extension at 72°C for 20 s. Relative changes in gene expressions were calculated using △△Ct (threshold cycle) method. The housekeeping genes such as B2M, ACTB, GAPDH, RPL27, HPRT1 and OAZ1 were used to normalize to the amount of RNA. Fold change values were calculated using the formula of 2-△△Ct.

### Cell extract preparation and western blot analysis

EV71-infected cells (MOI=5) were harvested as indicated. The pellet was washed and lysed with a lysis buffer (2% sodium dodecyl sulfate, 35 mM *β*-mercaptoethanol, 50 mM Tris–HCl [pH 6.8], 1 mM phenylmethylsulfonylfluoride). Cell lysates were obtained by centrifugation at 13000 rpm and 4°C. Total protein concentrations were determined by the bicinchoninic acid protein assay kit (Pierce). Then, proteins were resolved by sodium dodecyl sulfate polyacrylamide gel electrophoresis (SDS-PAGE), and transferred to PVDF membranes (Millipore). Membranes were blocked for 2 h with 5% nonfat dry milk solution in Tris-buffered saline containing 0.1% Tween-20, which were blotted with specific primary antibodies, followed by incubation with secondary antibodies conjugated with HRP. Blots were developed with an enhanced ChemiDoc™ XRS^+^ system (Biorad, USA).

### Statistical analysis

All data were presented as the mean ± SE. Statistical analyses were performed using GraphPad Prism software (San Diego, CA). P value less than 0.05 was considered as statistical significance.

## Results

### EV71 infection induced cytopathic effect in RD cells

**Figure 1:**
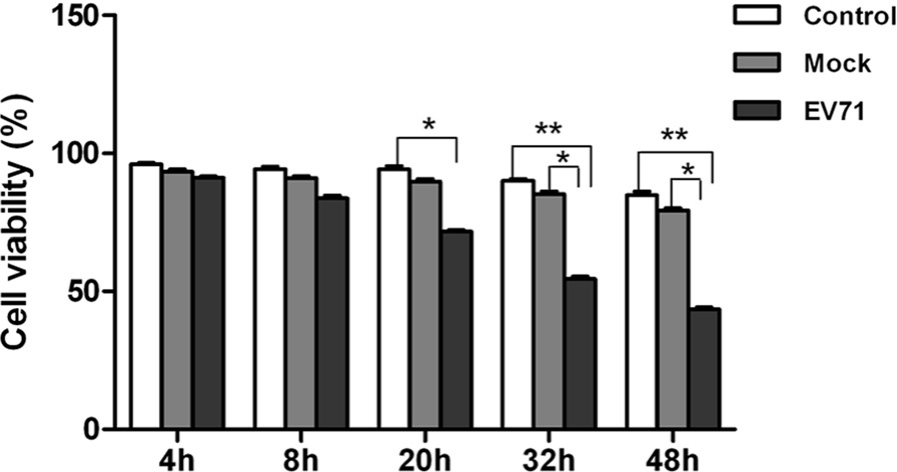
**The characteristics of EV71-infected RD cell proliferation in different time points.** Control: Uninfected RD cells. Mock: Inactivated EV71-infected RD cells. EV71: EV71-infected RD cells. Viable cell count was assessed by trypan blue exclusion technique. The data were expressed as mean ± SE from 3 independent experiments. * *P* < 0.05 and **P < 0.01 by two-way ANOVA.

**Figure 2:**
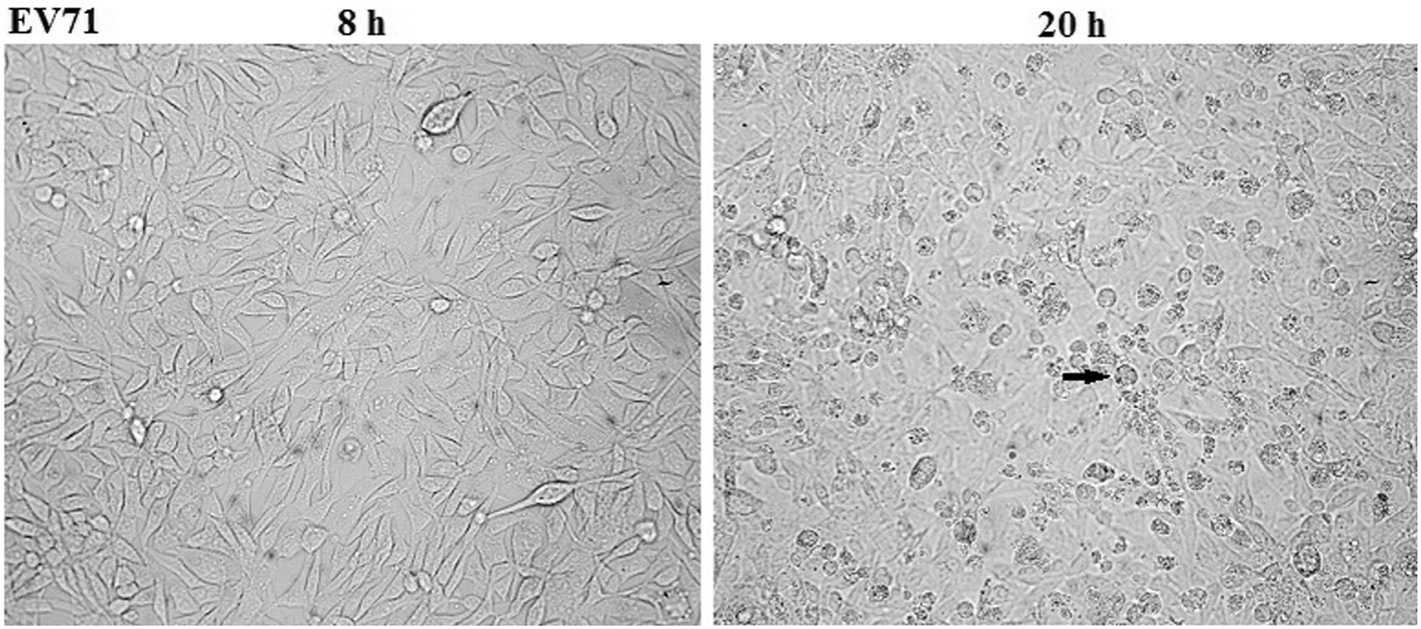
**The morphology of EV71-infected RD cells.** Following the infection at 8 and 20 h in RD cells, cell morphology was examined under a light microscope (100×), and visible cell apoptosis was labeled by black arrow.

Two batches of RD cells with three experimental models in each group were analyzed at 4, 8, 20, 32 and 48 h postinfection. Three models included uninfected control, mock-infection control with UV-inactivated EV71 strain GDV083 and EV71 strain GDV083 infection. The cell growth did not exhibit an obvious difference at 4 and 8 h postinfection. However, the viable cells revealed distinct decrease in EV71-infected model at 20, 32 and 48 h (Figure 1). Visible CPE was also observed in the infected cell culture (Figure 2). Viable cell counts for the uninfected and mock-infected controls were comparable without any potential “spillover” effects from the inoculum itself. In virus-infected cells, an obvious CPE was observed at 20 h postinfection. However, there was no significant change between mock control and uninfected control.

### Annexin V-FITC/PI fluorescent analysis of EV71-infected RD cells

**Figure 3:**
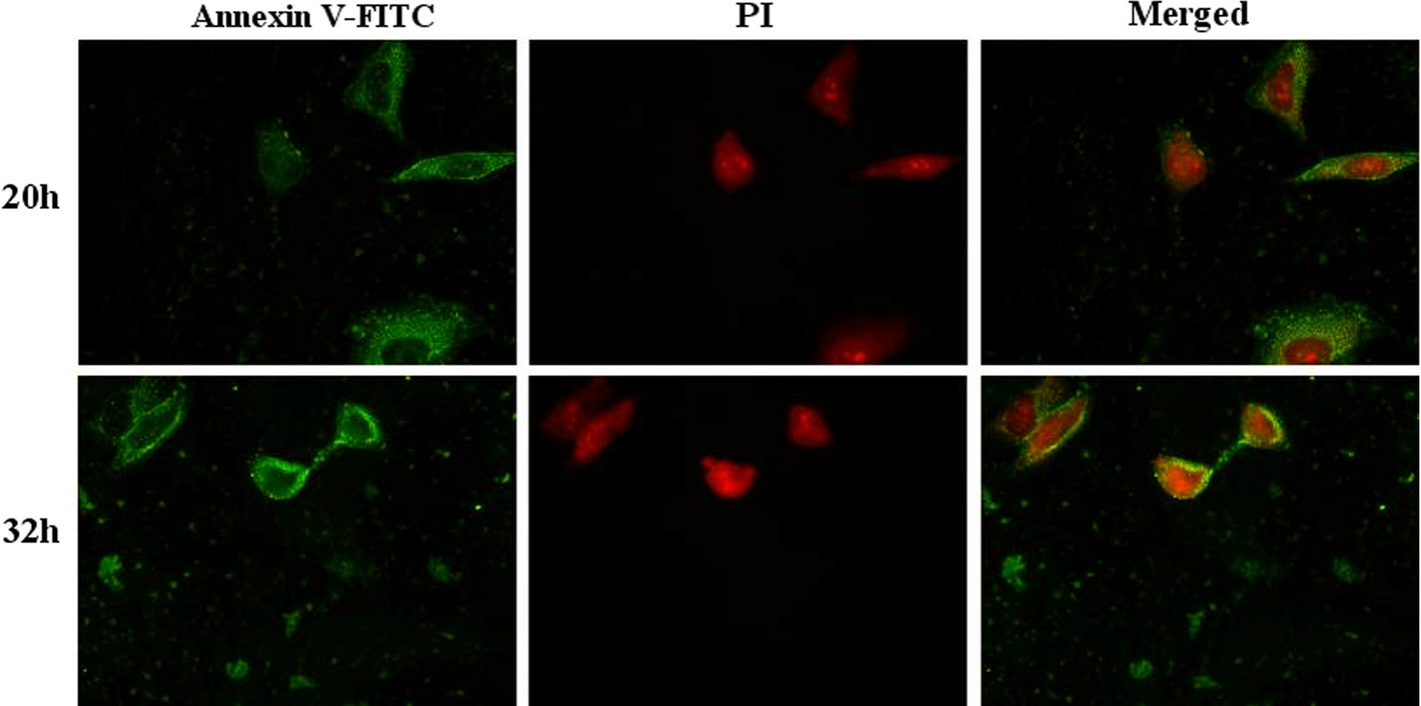
**EV71-induced apoptosis in RD cells.** Early membrane revealed an obvious change associated with apoptosis in EV71-infected RD cells. The exposure of phosphatidylserine (PS) was analyzed using FITC-labeled annexin V. Cell death was assessed with propidium iodide (PI). EV71-infected RD cells were harvested at 20 and 32 h postinfection and stained with annexin V-FITC/PI, and then observed under a fluorescence microscope (400×).

**Figure 4:**
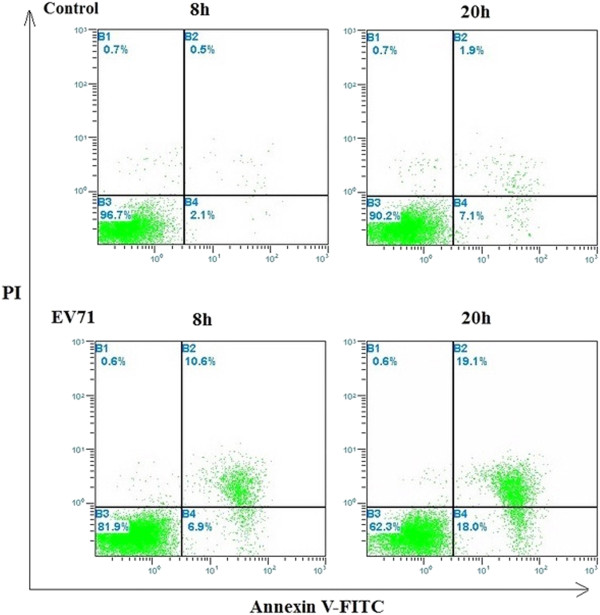
**Membrane changes associated with apoptosis in EV71-infected RD cells.** Control: Uninfected RD cells. EV71: EV71-infected RD cells. The exposure of phosphatidylserine (PS) was analyzed using FITC-labeled annexin V. Cell death was assessed using PI in a time course mode. EV71-infected RD cells were harvested at 8 and 20 h postinfection and cell death was evaluated by flow cytometry.

In normal cells, the phosphatidylserine (PS) was localized mainly on the membrane surface of cytoplasmic cells. However, PS could be translocated to the outer leaflet of the membrane during apoptosis. Dying cells stained with annexin V allowed for the detection of PS exposure as an early indicator of apoptosis. RD cells were infected by EV71 on glass coverslips at 20 h, which stained with annexin V-FITC/PI and visualized under a fluorescence microscope. RD cell apoptosis or death was observed at 20 and 32 h postinfection (Figure 3). Meanwhile, RD cells were infected with EV71 and membrane change was measured using flow cytometry. As shown in Figure 4, 6.9% of RD cells showed membrane change at 8 h, and 18% at 20 h. Moreover, cell death or late apoptosis was 10.6% and 19.1% at 8 and 20 h postinfection, respectively.

### Cytokine levels of IL-4, IL-10 and TNF-α

**Figure 5:**
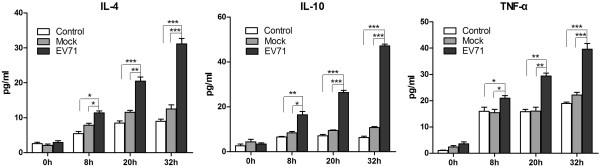
**EV71 infection activates the release of cytokines in RD cells.** The culture supernatants of control, mock and EV71-infected RD cells were harvested at 0, 8, 20 and 32 h after infection to measure the cytokines by ELISA. The data were expressed as mean ± SE of 3 independent experiments. *P < 0.05, **P < 0.01 and ***P < 0.001 by two-way ANOVA.

After EV71 infection at 0, 8, 20 and 32 h, the culture supernatants of RD cells were harvested, and then the levels of cytokines such as IL-4, IL-10 and TNF-α were detected by ELISA. The results suggested that EV71 infection could trigger RD cells to release IL-4, IL-10 and TNF-α (Figure 5).

### PCR array of 84 apoptotic genes in RD cells during EV71 infection

**Figure 6:**
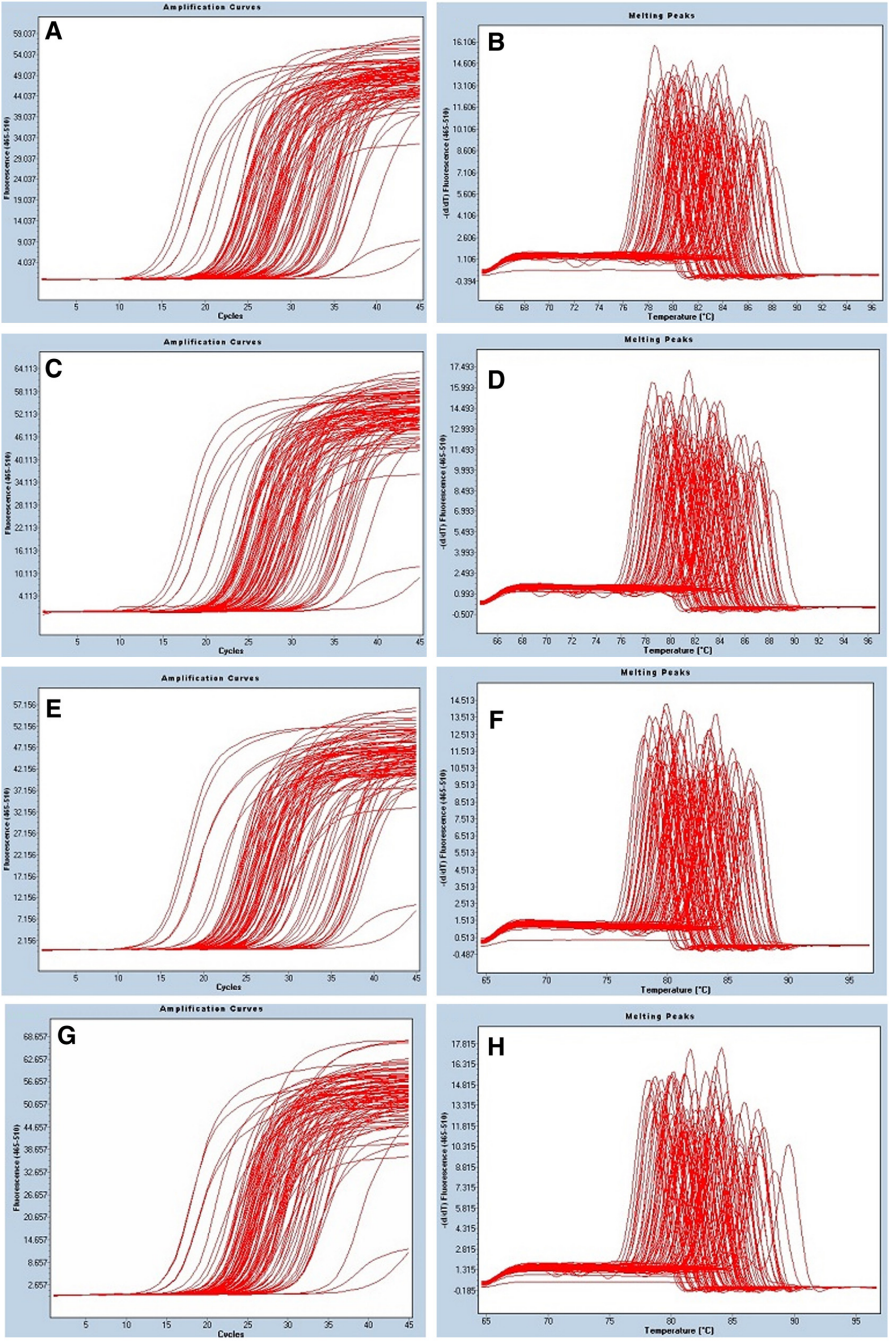
**PCR amplification and melting curves.** Uninfected Control: **A**, **B** and **E**, **F**; amplification curves and melting curves at 8 and 20 h, respecively. EV71-infected RD cells: **C**, **D** and **G**, **H**; amplification curves and melting curves at 8 and 20 h postinfection by EV71, respecively.

Cellular RNA was extracted and transcribed to cDNA in a 20 μL reaction mixture for 25 min at 40°C using random primers and SuperScript II reverse transcriptase (Invitrogen) according to the manufacturer’s instructions. The expression profile of 84 genes was generated. PCR amplification and melting curves were as shown in Figure 6. At 20 h postinfection, only 2 genes (c-Fos and IFN-β1) exhibited 2.34 and 5.22-fold increases, while 42 genes revealed a significant down-regulation. After EV71 infection at 20 h, the expression levels of 32 genes were larger than two fold based on PCR array. Particularly, the ligands of the TNF superfamily including FasL, CD40L and TNF-α were significantly up-regulated (Table 1).

**Table 1:** Differential apoptotic gene expressions of RD cells in response to EV71 infection at 8 and 20 h postinfection

**Symbols**	**Description of genes**	**EV71/control**	
		**(Fold changes)**	
		**8 h**	**20 h**
ACIN1	Apoptotic chromatin condensation inducer 1 (ACINUS; ACN; fSAP152)	-3.17	1.33
AIFM1	Apoptosis-inducing factor 1 (AIF; COXPD6; PDCD8)	1.03	-1.33
AIFM2	Apoptosis-inducing factor, mitochondrion-associated, 2 (AMID; PRG3; RP11-367H5.2)	-3.83	-1.25
Akt1	V-akt murine thymoma viral oncogene homolog 1 (AKT1; PKB; PKB-ALPHA; PRKBA; RAC; RAC-ALPHA)	-9.89	-1.13
Akt2	V-akt murine thymoma viral oncogene homolog 2 (AKT2, PKBB; PKBBETA; PRKBB; RAC-BETA)	-3.36	2.66
Akt3	V-akt murine thymoma viral oncogene homolog 3(AKT3)	-1.06	1.58
APAF1	Apoptotic peptidase activating factor 1 (APAF-1; CED4)	-4.10	1.09
API5	Apoptosis inhibitor 5 (AAC-11; AAC11)	-5.41	1.06
Bcl-2	B-cell lymphoma 2 (BCL-2)	-1.07	-1.43
BIRC2	Baculoviral IAP repeat-containing 2	1.56	1.23
BIRC3	Baculoviral IAP repeat-containing 3 (AIP1; API2; CIAP2; HAIP1; HIAP1; MALT2; MIHC; RNF49; c-IAP2)	-1.84	3.19
Caspase-3	Apoptosis-related cysteine peptidase 3(Casp3)	1.91	2.38
Caspase-4	Apoptosis-related cysteine peptidase 4(Casp4)	-1.31	1.15
Caspase-7	Apoptosis-related cysteine peptidase 7(Casp7)	-2.15	2.03
Caspase-8	Apoptosis-related cysteine peptidase 8(Casp8)	1.87	2.17
Caspase-9	Apoptosis-related cysteine peptidase 9(Casp9)	-4.98	-1.02
Caspase-10	Apoptosis-related cysteine peptidase 10(Casp10)	1.25	2.16
Caspase-12	Apoptosis-related cysteine peptidase 12 (Casp12)	1.24	1.10
CD5	CD5 molecule	-2.41	4.77
CD70	CD70 molecule	1.55	4.04
CD40L	CD40 ligand	-2.51	5.44
CD24	CD24 molecule	1.53	2.98
CD27	CD27 molecule	-2.37	1.71
c-Fos	c-fos proto-oncogene	2.34	3.23
c-Jun	c-Jun proto-oncogene	1.69	5.52
CIDEA	Cell death-inducing DFFA-like effector a	-1.32	-1.58
CIDEB	Cell death-inducing DFFA-like effector b	-2.67	2.98
DAPK1	Death-associated protein kinase 1	-2.80	1.24
DAPK2	Death-associated protein kinase 2	-1.81	1.32
DAPK3	Death-associated protein kinase 3 (ZIP; ZIPK)	-10.17	2.06
DFFA	DNA fragmentation factor subunit alpha (DFF-45; DFF1; ICAD)	-2.09	-1.14
E2F1	Transcription factor E2F1 (RBAP1; RBBP3; RBP3)	-3.99	-1.17
E2F2	Transcription factor E2F2	-1.64	1.55
EGFR	Epidermal growth factor receptor	-4.78	1.38
ENDOG	Endonuclease G	-11.37	1.06
ERBB3	Receptor tyrosine-protein kinase erbB-3 (ErbB-3; HER3; MDA-BF-1; c-erbB-3; p180-ErbB3; p45-sErbB3; p85-sErbB3)	-2.29	1.54
FADD	Fas-associated protein with death domain (MORT1)	-1.48	-1.42
FasL	Fas ligand (FAS L, CD95L; CD178; TNFSF6)	-1.14	7.53
Fas	Fas receptor (ALPS1A; APO-1; APT1; CD95; FAS1; FASTM; TNFRSF6)	-1.23	3.26
IFN-α2	Interferon alpha-2	1.13	1.54
IFN-β1	Interferon beta	5.22	1.47
IGF1	Insulin-like growth factor 1 (IGF-I; IGF1A; IGFI)	-1.14	-1.15
IGF1 R	Insulin-like Growth Factor 1 (IGF-1), Receptor (CD221; IGFIR; IGFR; JTK13)	-3.50	1.27
IKBKB	inhibitor of nuclear factor kappa-B kinase subunit beta (IKK-beta; IKK2; IKKB; NF-κBIKB)	-3.52	1.33
IKBKG	inhibitor of nuclear factor kappa-B kinase subunit gamma (AMCBX1; FIP3; IKK-gamma; IP1; IP2; IPD2; NEMO)	-5.76	1.40
IL-10	Interleukin 10	1.10	12.15
IL-1α	Interleukin-1 alpha	-2.60	1.64
IL-1β	Interleukin-1 beta	-2.20	1.30
IL-2	Interleukin 2	1.24	12.15
IL-4	Interleukin 4	-3.72	2.39
IL-6R	Interleukin 6 receptor (CD126)	-5.80	1.92
IL-7	Interleukin 7	-1.65	1.26
IRAK1	Interleukin-1 receptor-associated kinase 1	-3.70	3.39
JAK2	Janus kinase 2 (JTK10; THCYT3)	1.06	-1.21
MAP3K1	Mitogen-activated protein kinase kinase kinase 1 (MAPKKK1; MEKK; MEKK 1; MEKK1; SRXY6)	-4.55	1.51
MAP3K5	Mitogen-activated protein kinase kinase kinase 5 (ASK1; MAPKKK5; MEKK5)	-2.39	1.99
MAP2K4	Mitogen-activated protein kinase kinase 4 (JNKK1; MAPKK4; MEK4)	-1.06	2.54
MAP2K7	Mitogen-activated protein kinase kinase 7(MAPKK7; MEK7)	-1.09	3.05
MAPK1	Mitogen-activated protein kinase 1 (ERK; ERK2; ERT1; MAPK2; P42MAPK; PRKM1; PRKM2; p38; p40; p41)	-2.21	1.00
MAPK3	Mitogen-activated protein kinase 3(ERK1; HS44KDAP; P44ERK1; P44MAPK; PRKM3; p44-ERK1; p44-MAPK)	-2.74	1.67
MAPK8	Mitogen-activated protein kinase 8(JNK; JNK1; SAPK1)	1.73	2.53
MAPK9	Mitogen-activated protein kinase 9(JNK2; JNK2A; JNK2B; SAPK)	1.02	2.25
MYD88	Myeloid differentiation primary response gene (88)	-6.09	1.82
NF-κB1	Nuclear factor NF-kappa-B p105 subunit(NFKB-p105; NFKB-p50; NFkappaB; p105; p50)	-4.81	1.18
NF-κB3	Transcription factor p65 (RELA; NF-κB p65)	-1.35	2.63
NF-κBIA	Nuclear factor of kappa light polypeptide gene enhancer in B-cells inhibitor, alpha (IKBA; MAD-3; NFKBI)	1.49	2.96
NTRK1	Neurotrophic tyrosine kinase receptor type 1(MTC; TRK; TRK1; TRKA; Trk-A; p140-TrkA)	-2.84	1.33
PDCD1	Programmed cell death protein 1( CD279; PD-1; hPD-l)	-1.63	4.48
PDCD4	Programmed cell death protein 4	-1.30	1.06
PDCD 7	Programmed cell death protein 7	1.87	1.42
PI3K-α	Phosphoinositide-3-kinase, catalytic, alpha polypeptide(PIK3CA; p110-alpha; PI3K)	-1.26	1.04
PI3K-γ	Phosphatidylinositol-4,5-bisphosphate 3-kinase catalytic subunit gamma isoform (PIK3CG ; PI3K; PI3Kgamma; PIK3)	-1.40	5.18
PIK3R2	Phosphatidylinositol 3-kinase regulatory subunit beta(P85B; p85; p85-BETA)	-2.12	-1.20
PPP3R1	protein phosphatase 2B regulatory subunit 1(CALNB1; CNB; CNB1)	-1.52	1.94
RIPK1	Receptor-interacting serine/threonine-protein kinase 1(RIP; RIP1)	-2.44	1.24
STAT1	Signal transducers and activators of transcription (CANDF7; ISGF-3; STAT91)	-6.18	4.04
STAT5A	Signal transducer and activator of transcription 5A	-1.14	-1.15
STAT5B	Signal transducer and activator of transcription 5B	-3.17	1.08
TGFB1	Transforming growth factor beta 1(CED; DPD1; LAP; TGFB; TGFbeta)	-2.23	-1.15
TNF-α	Tumor necrosis factors	-1.97	2.19
TNFRSF10A	Tumor necrosis factor receptor superfamily member 10A (APO2; CD261; DR4; TRAILR-1; TRAILR1)	-2.82	2.03
TNFRSF10B	Tumor necrosis factor receptor superfamily member 10B (DR5, CD262, TRICK2, TRICKB, TRAILR2, TRICK2A, TRICK2B)	-2.84	1.02
P53	Tumor protein 53(LFS1; TP53; TRP53)	-7.60	1.32
XIAP	X-linked inhibitor of apoptosis protein(API3; BIRC4; IAP-3; ILP1; MIHA; XLP2; hIAP3)	-1.94	2.59

Negative values indicate down-regulation of genes.

### EV71 induced activation of death receptor signal pathway

**Figure 7:**
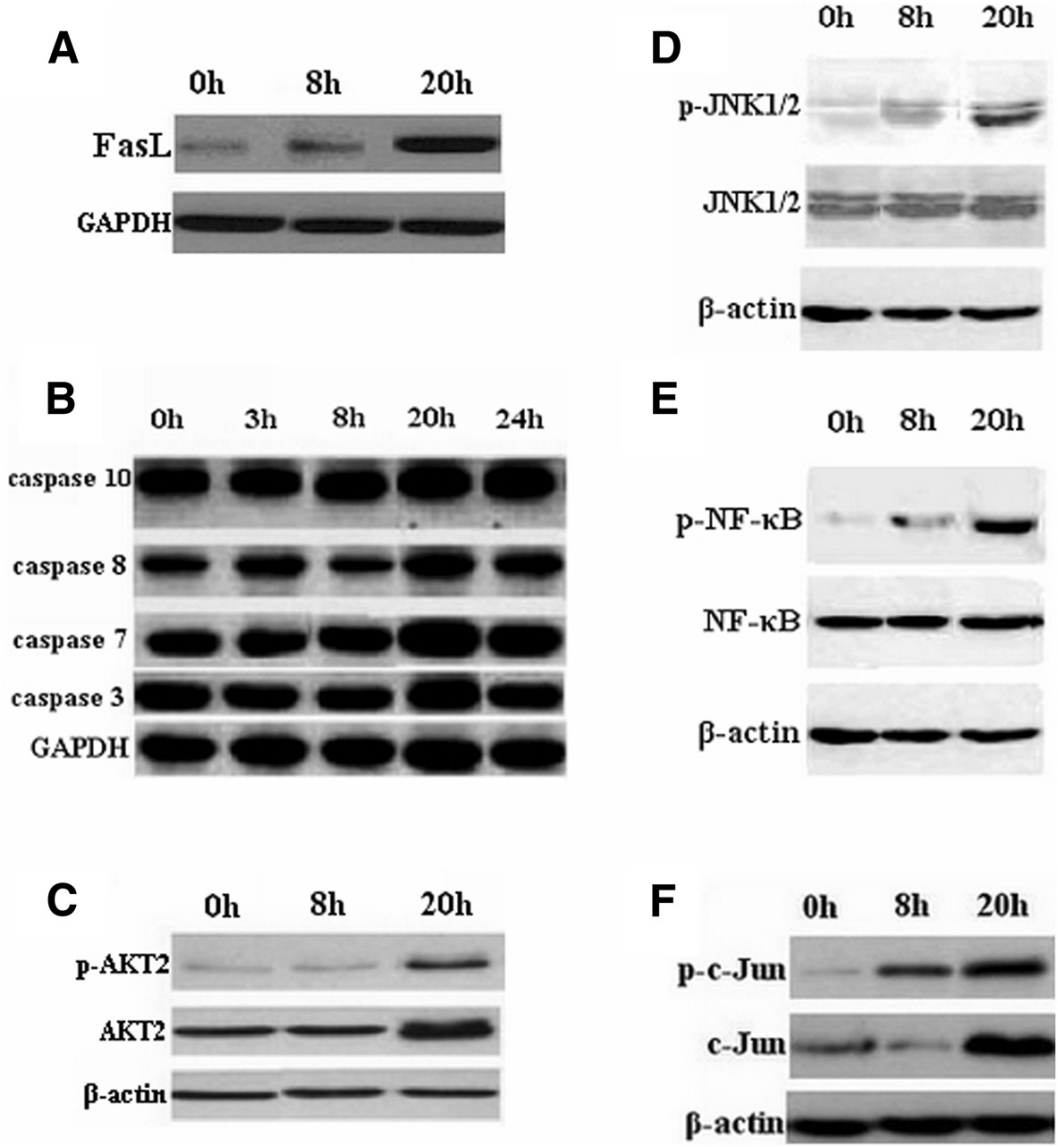
**EV71 infection induced activation of apoptotic signal pathway proteins.** Cell lysates were prepared from EV71-infected RD cells at the indicated time and resolved with 12% SDS-PAGE. Proteins were transferred onto PVDF membranes and subjected to western blotting. (**A**) FasL expression in EV71- infected RD cells at 0, 8, and 20 h. (**B**) Western blot analysis for caspase-10, -8, -7, and -3 in EV71- infected RD cells at 0, 3, 8, 20 and 24 h. GAPDH was probed as the loading control. The phosphorylated or total proteins of AKT2 (**C**), JNK1/2 (**D**), NF-κB (**E**) and c-Jun (**F**) were detected by western blotting at 0, 8, and 20 h. The amounts of β-actin were also assessed to monitor the equal loadings of protein extracts.

**Figure 8:**
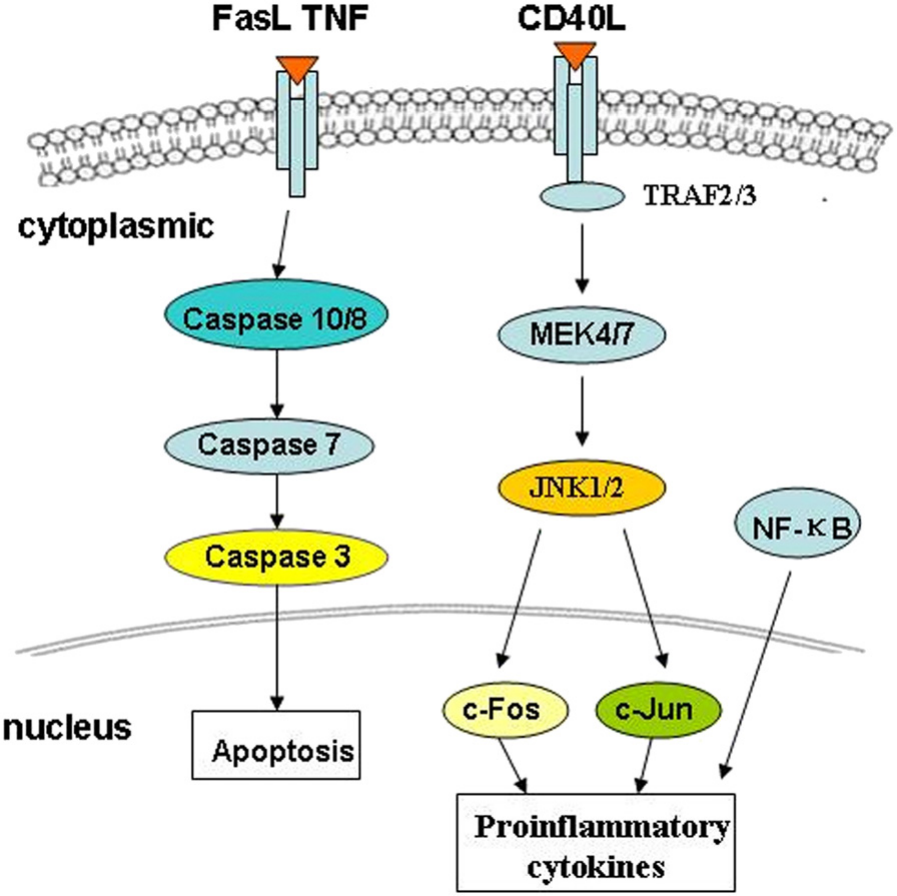
**Putative apoptotic pathways in EV71-infected RD cells.** The pathways were deduced from PCR array and western blotting analysis.

When RD cells were infected with EV71 at 20 h, the expressions of caspase -10, -8, -7, -3 and FasL were highly elevated. Upon death ligand-receptor binding, caspase-10 is coupled to the multimeric Fas/TNF receptor complex via DED/FADD adaptor interaction. This complex may process procaspase-10 and -8 into a large active subunit and a small subunit. Then cleaved caspase-10 and -8 can further process other caspase members, including caspase-7 and -3, to initiate a caspase cascade, leading to programmed cell death. Interestingly, the gene expressions of CD40L, MEK4, MEK7 and c-Fos were induced by 5.44, 2.54, 3.05 and 3.23 fold in RD cells at 20 h posinfection. In addition, JNK1/2, NF-κB p65 and c-Jun were significantly activated and phosphorylated (Figure 7). Based on these results, putative pathways of apoptosis were predicted for EV71-infected RD cells (Figure 8).

## Discussion

Apoptosis is characterized by DNA fragmentation, chromatin condensation, membrane blebbing, cell shrinkage and cell death. An increasing number of viruses can induce apoptosis at the late stage of infection. This process may be important for the spread of progeny viruses to the neighbor cells and for the protection of progeny viruses against host enzymes and antibodies. There are three major apoptotic pathways including death receptor (extrinsic), mitochondrial (intrinsic) and endoplasmic reticulum pathways, respectively. All of these pathways are observed in the cytoplasm [21,22]. The mechanisms of host defense can limit virus replication, enable infected cells apoptosis, release and exclude immature virus particles through lysosome or proteases. However, the viruses survive through a variety of ways which can interfere with the apoptosis of infected cells to evade or delay the early onset of apoptosis [16,23].

In order to confirm the pathophysiology of EV71 infection and host cellular responses, especially for apoptotic responses, we have employed PCR to analyze the expression changes of cellular genes during the infection process. The expression profiles of 84 pro- and anti-apoptotic genes revealed a significant difference in various time points. With the onset of EV71 replication, the genes such as ACIN1, Akt, APAF1, caspase, CIDEB, DAPK, NF-κB1, STAT1 and p53 associated with apoptosis were interrupted at 8 h postinfection by apoptosis-suppressing effect of EV71. Although many RNA viruses do not encode anti-apoptotic genes, the apoptosis-suppressing mechanisms are still unclear. EV71 infection at 20 h postinfection could stimulate the up-regulation of genes such as FasL, CD40L, TNF-α, TNFRSF10A (DR4), Fas, caspase-10, -8, -7, -3 and XIAP involved in TNFR1 pathway in RD cells. FasL is a type II membrane protein in the TNF-α receptor family and Fas receptor binding with FasL can induce cell apoptosis [18,24]. Interestingly, the expressions of Fas, FasL and DR4 revealed an enhancement by 3.26, 7.53 and 2.03 fold in RD cells at 20 h postinfection, suggesting that the proteins involved in Fas receptor-mediated pathway were activated in EV71-infected RD cells. Furthermore, downstream proteins in the Fas pathway such as caspase-10, -8, -7, -3 and CIDEB were also significantly up-regulated. The activation of TNFR1, CD95 and DR4/DR5 by TNF-α, FasL and TRAIL can trigger the activation of caspase-10, -8, -7, and -3 involved in cell death. The activated caspase-3 can cleave its substrates such as CIDE-B to trigger chromosomal DNA fragmentation. However, the expressions of caspase-12, -9, Bcl-2, and APAF1 were unchanged in EV71-infected cells. Previous studies reported that the death receptor pathway for apoptosis was the major pathway during the EV71 infection of RD cells [22,25]. In this study, differential changes of apoptotic gene expressions may be attributed to different conditions such as MOI, infection duration and EV71 strains.

CD40L is described originally in T lymphocytes. However, its expression has been observed in a wide variety of cells including platelets, mast cells, macrophages, basophils, NK cells, B lymphocytes as well as non-haematopoietic cells [26]. As a member of the TNF-α receptor family, CD40 relies on interaction with TRAF proteins to mediate an intracellular signal in response to CD40L binding [27,28]. The downstream protein of TRAF2/3 can activate consecutive initiation of MEK4 and MEK7, resulting in the activation of stress-activated protein kinases JNK1/2 [29]. Subsequently, transcription factor activator of proteins (c-Fos and c-Jun) and NF-κB were activated through a kinase pathway involving in map kinases, NIK (NF-κB inducing kinase) and I-κB kinase, thus co-stimulating the proliferation of activated T-cells accompanied by the production of IFN-γ, TNF-α, and IL-2 [28]. Interestingly, the expressions of CD40L, MEK4 and MEK7 were induced by 5.44, 2.54, and 3.05-fold in RD cells at 20 h postinfection, whereas JNK1/2 was highly phosphorylated. Therefore, we postulated that CD40L/CD40 signaling pathway was also activated in EV71-infected RD cells.

The serine/threonine kinase AKT, also known as protein kinase B (PKB), has become a major focus because of its critical regulatory role in cellular processes including cancer progression [30]. AKT cascades are activated by receptor tyrosine kinases, integrins, B- and T-cell receptors, cytokine receptors, G-protein coupled receptors and other stimuli for inducing the accumulation of phosphatidylinositol 3,4,5-triphosphates by phosphoinositide 3-kinase (PI3K) [31]. Three AKT isoforms (AKT1, AKT2 and AKT3) can mediate many downstream events regulated by PI3K. At 8 h postinfection, the expression of AKT2 was significantly down-regulated by 3.36 fold, while PI3K and AKT2 were enhanced by 5.18 fold and 2.66 fold at 20 h postinfection, respectively. The phosphorylation of AKT2 was highly elevated. As previously reported, EV71 can activate PI3K/AKT to trigger the anti-apoptotic pathways at the early phase during infection [32,33]. In this study, the activation of PI3K/AKT may be associated with EV71 strain virulence and virus titer.

Transcription factor NF-κB p65 (NF-κB3) is a protein encoded *RELA* gene in human. Activated NF-κB p65 translocates into the nucleus and binds DNA at kappa-B-binding motifs, thus being involved in the production of cytokines including IL-2, IL-6, TNF-α, IFN-β and IL-1β [34,35]. While STAT1 is a member of the signal transducers and activators of transcription family and involved in the up-regulation of the genes through type I, type II or type III interferon [36]. Both NF-κB and STAT1 are rapidly activated in response to various stimuli including viral infection and cytokines, thus controlling the expressions of anti-apoptotic, pro-proliferative and immune response genes. In this study, NF-κB p65 and STAT1 were upregulated by 2.63 and 4.04 fold, respectively. Meanwhile, NF-κB p65 was significantly phosphorylated at 20 h postinfection. However, NF-κBIA was up-regulated by 2.96 fold, which can block NF-κB to bind to DNA. IL-2, IL-4, IL-10 and TNF-α exhibited the enhancement by 12.15, 2.39, 12.15 and 2.19 fold at 20 h after EV71 infection. Compared with EV71-uninfected controls, the expression levels of IL-4, IL-10 and TNF-α in EV71-infected RD cells revealed a significant increase at 20 and 32 h postinfection (*P* < 0.01,or *P* < 0.001).

Eight human IAPs such as NAIP (BIRC1), c-IAP1 (BIRC2), c-IAP2 (BIRC3), X-linked IAP (XIAP, BIRC4), Survivin (BIRC5), Apollon (BRUCE, BIRC6), Livin/ML-IAP (BIRC7) and IAP-like protein 2 (BIRC8) have been identified [37,38]. BIRC3 can participate in TNF-α-mediated NF-κB activation, while XIAP can bind to the active site of effector caspases including caspase-3, caspase-7 and caspase-9 for preventing substrate binding and subsequent catalysis through its BIR2 domain with N-terminal linker [39]. BIRC3 and XIAP were significantly up-regulated by 3.19 fold and 2.59 fold at 20 h postinfection, which may be related to the apoptosis inhibition of RD cells. Additionally, the up-regulation of CD5, CD24, CD70, CD226 and PDCD1 (PD-1) that usually express on the surface of T cells, B cells, dendritic cells, NK cells, and tumor cells have also been observed [40-44]. Interestingly, these genes revealed a significant up-regulation in EV71-infected RD cells and may be associated with cell signal pathway and apoptosis. Therefore, further study is needed to be undertaken for these gene expressions.

## Conclusions

PCR array is an effective method to analyze differential gene expressions on cellular signaling pathways. The results showed that RD cells have a general trend to inhibit cell proliferation and delay the apoptotic process during the early stage of EV71 infection. EV71 induced-apoptosis may be associated with the activation of Fas, CD40 and TNF death receptor as well as the cleavage of initiator caspases including caspase-10 and -8. However, the activation of caspase-12, -9 and APAF1 in mitochondrial apoptotic pathway and endoplasmic reticulum stress was not completed in EV71-infected RD cells, which may be associated with MOI, infection duration and EV71 strains, etc. In response to EV71 infection, both NF-κB and c-Jun are activated to translocate into the nucleus to induce the production of IL-4, IL-10 and TNF-α. Thus, our findings will provide a theoretical evidence for reducing cell damage and intervening EV71 infection process.

## Competing interests

The authors declare that they have no competing interests.

## Authors’ contributions

WS designed the experiments, and participated in data analysis and manuscript writing. XL, XH and HP performed RD cell culture, flow cytometry analysis and PCR. QJ participated in coordination and partial data analysis of experiments. MS and YJ performed cell viability counts and partial data analysis. XL and JL designed ELISA assays and the detection of cytokines. All authors reviewed and approved the final manuscript.

## Pre-publication history

The pre-publication history for this paper can be accessed here:

http://www.biomedcentral.com/1471-2334/12/327/prepub

## References

[B1] Yan JJ Su IJ Chen PF Liu CC Yu CK Wang JR Complete genome analysis of enterovirus 71 isolated from an outbreak in Taiwan and rapid identification of enterovirus 71 and coxsackievirus A16 by RT-PCR J Med Virol 2001 65 331 339 10.1002/jmv.2038 11536241

[B2] Brown BA Pallansch MA Complete nucleotide sequence of enterovirus 71 is distinct from poliovirus Virus Res 1995 39 195 205 10.1016/0168-1702(95)00087-9 8837884

[B3] Zhang Y Tan XJ Wang HY Yan DM Zhu SL Wang DY Ji F Wang XJ Gao YJ Chen L An HQ Li DX Wang SW Xu AQ Wang ZJ Xu WB An outbreak of hand, foot, and mouth disease associated with subgenotype C4 of human enterovirus 71 in Shandong, China J Clin Virol 2009 44 262 267 10.1016/j.jcv.2009.02.002 19269888

[B4] Chang LY Lin TY Hsu KH Huang YC Lin KL Hsueh C Shih SR Ning HC Hwang MS Wang HS Lee CY Clinical features and risk factors of pulmonary oedema after enterovirus-71-related hand, foot, and mouth disease Lancet 1999 354 1682 1686 10.1016/S0140-6736(99)04434-7 10568570

[B5] Lum LC Wong KT Lam SK Chua KB Goh AY Lim WL Ong BB Paul G AbuBakar S Lambert M Fatal enterovirus 71 encephalomyelitis J Pediatr 1998 133 795 798 10.1016/S0022-3476(98)70155-6 9842048

[B6] Schmidt NJ Lennette EH Ho HH An apparently new enterovirus isolated from patients with disease of the central nervous system J Infect Dis 1974 129 304 309 10.1093/infdis/129.3.304 4361245

[B7] Wang SM Liu CC Tseng HW Wang JR Huang CC Chen YJ Yang YJ Lin SJ Yeh TF Clinical spectrum of enterovirus 71 infection in children in southern Taiwan, with an emphasis on neurological complications Clin Infect Dis 1999 29 184 190 10.1086/520149 10433583

[B8] McMinn P Lindsay K Perera D Chan HM Chan KP Cardosa MJ Phylogenetic analysis of enterovirus 71 strains isolated during linked epidemics in Malaysia, Singapore, and Western Australia J Virol 2001 75 7732 7738 10.1128/JVI.75.16.7732-7738.2001 11462047 PMC115010

[B9] Ma E Chan KC Cheng P Wong C Chuang SK The enterovirus 71 epidemic in 2008-public health implications for Hong Kong Int J Infect Dis 2010 14 e775 e780 10.1016/j.ijid.2010.02.2265 20599410

[B10] Li L He Y Yang H Zhu J Xu X Dong J Zhu Y Jin Q Genetic characteristics of human enterovirus 71 and coxsackievirus A16 circulating from 1999 to 2004 in Shenzhen, People's Republic of China J Clin Microbiol 2005 43 3835 3839 10.1128/JCM.43.8.3835-3839.2005 16081920 PMC1233905

[B11] Chua KB Kasri AR Hand foot and mouth disease due to enterovirus 71 in Malaysia Virol Sin 2011 26 221 228 10.1007/s12250-011-3195-8 21847753 PMC8222466

[B12] Shore GC Papa FR Oakes SA Signaling cell death from the endoplasmic reticulum stress response Curr Opin Cell Biol 2011 23 143 149 10.1016/j.ceb.2010.11.003 21146390 PMC3078187

[B13] Krampe B Al-Rubeai M Cell death in mammalian cell culture: molecular mechanisms and cell line engineering strategies Cytotechnology 2010 62 175 188 10.1007/s10616-010-9274-0 20502964 PMC2932912

[B14] Leong WF Chow VT Transcriptomic and proteomic analyses of rhabdomyosarcoma cells reveal differential cellular gene expression in response to enterovirus 71 infection Cell Microbiol 2006 8 565 580 10.1111/j.1462-5822.2005.00644.x 16548883 PMC7162300

[B15] Clem R Miller L Apoptosis reduces both the in vitro replication and the in vivo infectivity of a baculovirus J Virol 1993 67 3730 3738 8510202 10.1128/jvi.67.7.3730-3738.1993 PMC237736

[B16] Sun E Shi Y Apoptosis: the quiet death silences the immune system Pharmacol Ther 2001 92 135 145 10.1016/S0163-7258(01)00164-4 11916534

[B17] Liang CC Sun MJ Lei HY Chen SH Yu CK Liu CC Wang JR Yeh TM Human endothelial cell activation and apoptosis induced by enterovirus 71 infection J Med Virol 2004 74 597 603 10.1002/jmv.20216 15484266

[B18] Chen LC Shyu HW Chen SH Lei HY Yu CK Yeh TM Enterovirus 71 infection induces Fas ligand expression and apoptosis of Jurkat cells J Med Virol 2006 78 780 786 10.1002/jmv.20623 16628611

[B19] Weng KF Chen LL Huang PN Shih SR Neural pathogenesis of enterovirus 71 infection Microbes Infect 2010 12 505 510 10.1016/j.micinf.2010.03.006 20348010

[B20] Hung HC Chen TC Fang MY Yen KJ Shih SR Hsu JT Tseng CP Inhibition of enterovirus 71 replication and the viral 3D polymerase by aurintricarboxylic acid J Antimicrob Chemother 2010 65 676 683 10.1093/jac/dkp502 20089540 PMC7110181

[B21] Szegezdi E Macdonald DC Ni-Chonghaile T Gupta S Samali A Bcl-2 family on guard at the ER Am J Physiol Cell Physiol 2009 296 C941 C953 10.1152/ajpcell.00612.2008 19279228

[B22] Chang SC Lin JY Lo LY Li ML Shih SR Diverse apoptotic pathways in enterovirus 71-infected cells J Neurovirol 2004 10 338 349 10.1080/13550280490521032 15765805

[B23] Abendroth A Kinchington PR Slobedman B Varicella zoster virus immune evasion strategies Curr Top Microbiol Immunol 2010 342 155 171 10.1007/82_2010_41 20563710 PMC3936337

[B24] Lin YW Wang SW Tung YY Chen SH Enterovirus 71 infection of human dendritic cells Exp Biol Med (Maywood) 2009 234 1166 1173 10.3181/0903-RM-116 19596831

[B25] Farley SM Purdy DE Ryabinina OP Schneider P Magun BE Iordanov MS Fas ligand-induced proinflammatory transcriptional responses in reconstructed human epidermis. Recruitment of the epidermal growth factor receptor and activation of MAP kinases J Biol Chem 2008 283 919 928 10.1074/jbc.M705852200 17977827

[B26] Lee VW Qin X Wang Y Zheng G Ince J Tan TK Kairaitis LK Alexander SI Harris DC The CD40-CD154 co-stimulation pathway mediates innate immune injury in adriamycin nephrosis Nephrol Dial Transplant 2010 25 717 730 10.1093/ndt/gfp569 19889873

[B27] Jundi M Nadiri A Al-Zoobi L Hassan GS Mourad W CD40-mediated cell death requires TRAF6 recruitment Immunobiology 2012 217 375 383 10.1016/j.imbio.2011.07.007 21813202

[B28] Elgueta R Benson MJ de Vries VC Wasiuk A Guo Y Noelle RJ Molecular mechanism and function of CD40/CD40L engagement in the immune system Immunol Rev 2009 229 152 172 10.1111/j.1600-065X.2009.00782.x 19426221 PMC3826168

[B29] Rau SJ Hildt E Himmelsbach K Thimme R Wakita T Blum HE Fischer R CD40 inhibits replication of hepatitis C virus in primary human hepatocytes by JNK activation independent from the interferon pathway Hepatology 2012 10.1002/hep.25966 22814930

[B30] Dillon RL Muller WJ Distinct biological roles for the akt family in mammary tumor progression Cancer Res 2010 70 4260 4264 10.1158/0008-5472.CAN-10-0266 20424120 PMC2880222

[B31] Tong KM Shieh DC Chen CP Tzeng CY Wang SP Huang KC Chiu YC Fong YC Tang CH Leptin induces IL-8 expression via leptin receptor, IRS-1, PI3K, Akt cascade and promotion of NF-kappaB/p300 binding in human synovial fibroblasts Cell Signal 2008 20 1478 1488 10.1016/j.cellsig.2008.04.003 18501560

[B32] Wong WR Chen YY Yang SM Chen YL Horng JT Phosphorylation of PI3K/Akt and MAPK/ERK in an early entry step of enterovirus 71 Life Sci 2005 78 82 90 10.1016/j.lfs.2005.04.076 16150462 PMC7094582

[B33] Autret A Martin-Latil S Brisac C Mousson L Colbere-Garapin F Blondel B Early phosphatidylinositol 3-kinase/Akt pathway activation limits poliovirus-induced JNK-mediated cell death J Virol 2008 82 3796 3802 10.1128/JVI.02020-07 18216097 PMC2268503

[B34] Piva R Belardo G Santoro MG NF-kappaB: a stress-regulated switch for cell survival Antioxid Redox Signal 2006 8 478 486 10.1089/ars.2006.8.478 16677091

[B35] Mauro C Zazzeroni F Papa S Bubici C Franzoso G The NF-kappaB transcription factor pathway as a therapeutic target in cancer: methods for detection of NF-kappaB activity Methods Mol Biol 2009 512 169 207 10.1007/978-1-60327-530-9_10 19347278

[B36] Sow FB Alvarez GR Gross RP Satoskar AR Schlesinger LS Zwilling BS Lafuse WP Role of STAT1, NF-kappaB, and C/EBPbeta in the macrophage transcriptional regulation of hepcidin by mycobacterial infection and IFN-gamma J Leukoc Biol 2009 86 1247 1258 10.1189/jlb.1208719 19652026

[B37] Gill C Dowling C O'Neill AJ Watson RW Effects of cIAP-1, cIAP-2 and XIAP triple knockdown on prostate cancer cell susceptibility to apoptosis, cell survival and proliferation Mol Cancer 2009 8 39 10.1186/1476-4598-8-39 19549337 PMC2706796

[B38] Augello C Caruso L Maggioni M Donadon M Montorsi M Santambrogio R Torzilli G Vaira V Pellegrini C Roncalli M Coggi G Bosari S Inhibitors of apoptosis proteins (IAPs) expression and their prognostic significance in hepatocellular carcinoma BMC Cancer 2009 9 125 10.1186/1471-2407-9-125 19397802 PMC2680906

[B39] Jin HS Lee DH Kim DH Chung JH Lee SJ Lee TH cIAP1, cIAP2, and XIAP act cooperatively via nonredundant pathways to regulate genotoxic stress-induced nuclear factor-kappaB activation Cancer Res 2009 69 1782 1791 10.1158/0008-5472.CAN-08-2256 19223549

[B40] Dalloul A CD5: a safeguard against autoimmunity and a shield for cancer cells Autoimmun Rev 2009 8 349 353 10.1016/j.autrev.2008.11.007 19041428

[B41] Minton K T cell memory: Exhausted T cells miss out on methylation Nat Rev Immunol 2011 11 718 719 21997790 10.1038/nri3093

[B42] Overdevest JB Thomas S Kristiansen G Hansel DE Smith SC Theodorescu D CD24 offers a therapeutic target for control of bladder cancer metastasis based on a requirement for lung colonization Cancer Res 2011 71 3802 3811 10.1158/0008-5472.CAN-11-0519 21482678 PMC4283788

[B43] Zwart W Peperzak V de Vries E Keller AM van der Horst G Veraar EAM Geumann U Janssen H Janssen L Naik SH The invariant chain transports TNF family member CD70 to MHC class II compartments in dendritic cells J Cell Sci 2010 123 3817 3827 10.1242/jcs.068510 20971706

[B44] Tahara-Hanaoka S Shibuya K Onoda Y Zhang H Yamazaki S Miyamoto A Honda S Lanier LL Shibuya A Functional characterization of DNAM-1 (CD226) interaction with its ligands PVR (CD155) and nectin-2 (PRR-2/CD112) Int Immunol 2004 16 533 538 10.1093/intimm/dxh059 15039383

